# Supplementary material for: Human brain representations of internally generated outcomes of approximate calculation revealed by ultra-high-field brain imaging
Source: Nat Commun. 2024 Jan 17;15:572. doi: 10.1038/s41467-024-44810-5 (PMC10794709; doi:10.1038/s41467-024-44810-5)
Supplement: Supplementary file 1 — Supplementary Information [file 41467_2024_44810_MOESM1_ESM.pdf]

# Supplementary Information for Human brain representations of internally generated outcomes of approximate calculation revealed by ultra-high-field brain imaging

Supplementary Methods:

To explore effects of the different experimental effects of interest (sample numerosity, operation, operand, result numerosity) on brain activity in univariate analyses using standard models of the hemodynamic response, an additional first level model of the fMRI data was computed in each subject. This model included the following  set of predictors for the standard trial types (catch trials being modelled separately and not included in the analysis): 4 predictors for the 4 different sample numerosities (6, 12, 24, 48) with stimulus onset at the time of sample display appearance, 2 predictors for operands (2 and 4), timed at the onset of the operation instruction display, two predictors for the operations (multiplication and division) with their onset defined at 500 ms after the operation instruction display, 4 predictors for the possible result numerosities (6, 12, 24, 48) with their onset defined at 1s after the operation instruction display, and 8 predictors for the match numerosity (separated into smaller and larger for each of the four results) at the onset of the match display. All predictor were modelled as stick functions convolved with the canonical hemodynamic response function of SPM12. The GLM included the six motion parameters as covariate of no interest, an AR(1) model was used to account for serial auto-correlation and low-frequency signal drifts were removed by a high-pass filter with a cutoff of 244 s. The following contrasts were created at the individual subject level: The parametric increase with numerosity, separately for the sample and result numerosity (contrast weights -1.5 -0.5 0.5 1.5) and differential contrasts for each individual numerosity vs all others, separately for the sample and result (e.g., contrast weights [3 -1 -1 -1] for numerosity 6). For each of these, second level analyses were then conducted using one-sample t-tests in Freesurfer (after projecting all contrasts into FsAverage space). Results are presented in Supplementary Figure 1 and Supplementary Table 1.

To test for effects of probe ratio on BOLD responses, an additional FIR model was fitted where the conditions were redefined as a function of probe ratio.  This model included 4 predictors corresponding to the four levels of ratio between probe and result while collapsing probe larger and probe smaller trials for each level, and an additional predictor including all catch trials. Effects of probe ratio are expected during the part of the trial after probe appearance when subjects carry out the comparison task, and results can serve as a sanity check testing for whether effects related to this factor are indeed absent from the data during other periods of the trial (i.e., do not affect the following trial's delay period in any systematic way). Contrasts grouped together the two smallest and two largest ratios, and results for the contrast (close > far ratio) corresponding to a classical comparison distance effect as often reported in the literature, are reported in Supplementary Figure 6 and Supplementary Table 5.

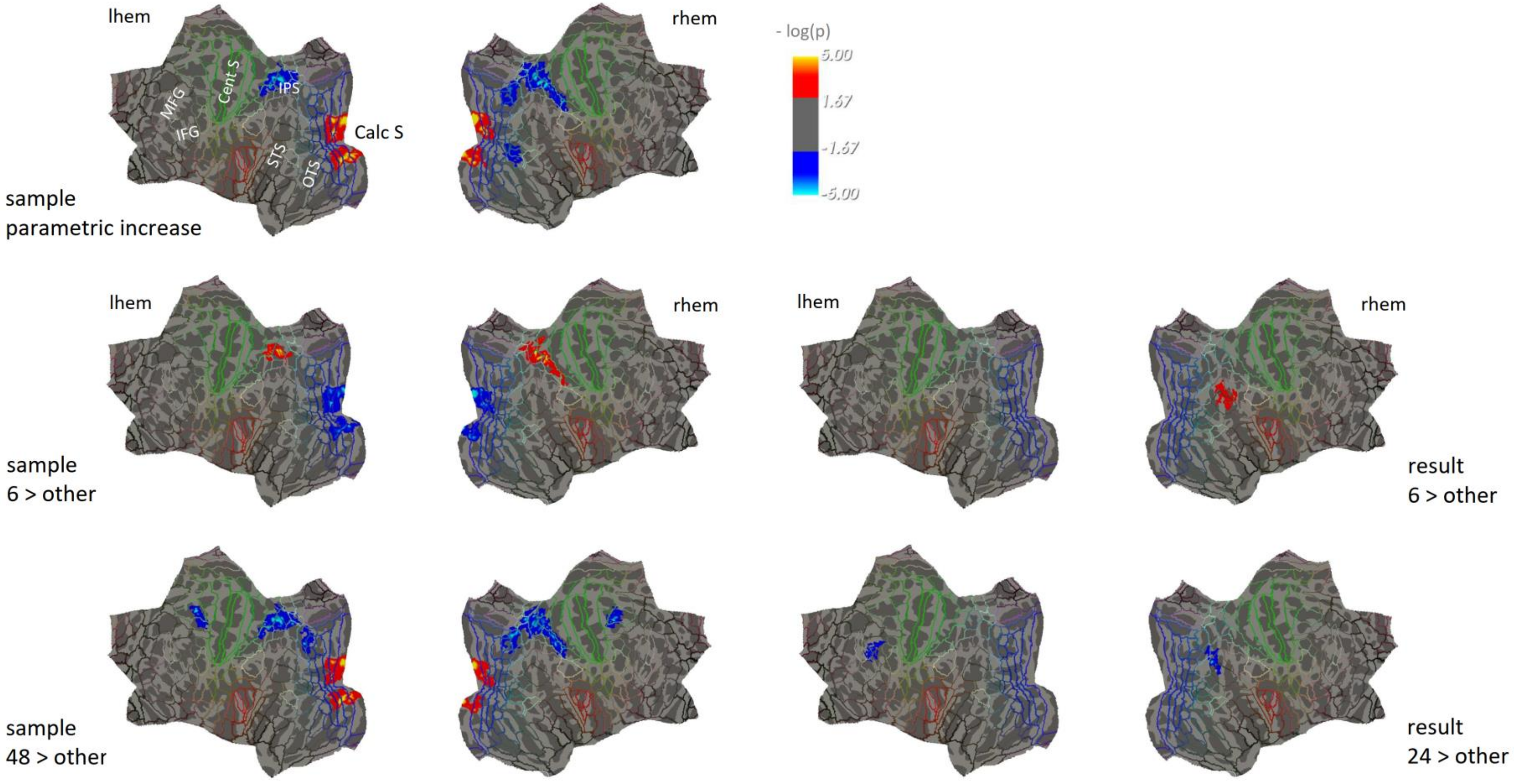


**Supplementary Figure 1: Parametric increases with numerosity and differential effects of each specific numerosity vs all others**. Random effects group analyses (n = 17 subjects) were performed on univariate brain responses in surface space using FreeSurfer (two-tailed one-sample t-tests, correction for multiple comparisons by permutation at cluster level, pFWE <.05, cluster forming threshold p < .01). The color scale represents uncorrected -log(p) values within the clusters surviving correction. Results are projected onto a flattened cortical average surface (https://mri.sdsu.edu/sereno/csurf/), with superimposed lines representing the regional borders of the Human Connectome Project (HCP-MMP1) parcellation. For better orientation, the location of major anatomical landmarks (MFG: middle frontal gyrus, IFG: inferior frontal gyrus, Cent S: central sulcus, IPS: intraparietal sulcus, STS: superior temporal sulcus, OTS: occipitotemporal sulcus, Calc S: calcarine sulcus) is indicated. Source data are provided as a Source Data file.

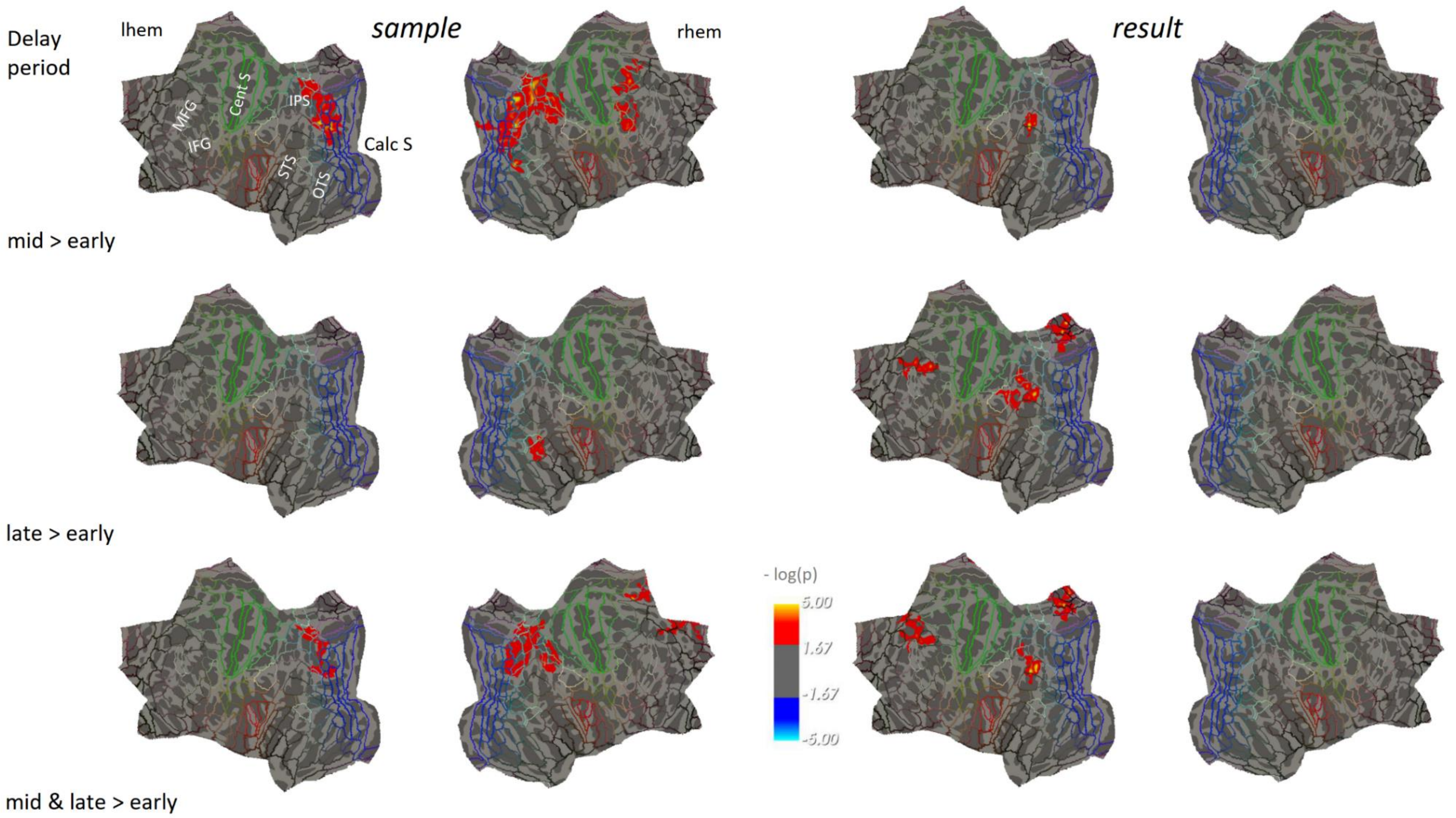


**Supplementary Figure 2: Differential effects between time windows in multiple regression-based representational similarity analysis.** Random effects group analyses (n = 17 subjects) were performed in surface space using FreeSurfer (two-tailed one-sample t-tests, correction for multiple comparisons by permutation at cluster level, pFWE <.05, cluster forming threshold p < .01). The color scale represents uncorrected -log(p) values within the clusters surviving correction. Results are projected onto a flattened cortical average surface (https://mri.sdsu.edu/sereno/csurf/), with superimposed lines representing the regional borders of the Human Connectome Project (HCP-MMP1) parcellation. For better orientation, the location of major anatomical landmarks (MFG: middle frontal gyrus, IFG: inferior frontal gyrus, Cent S: central sulcus, IPS: intraparietal sulcus, STS: superior temporal sulcus, OTS: occipitotemporal sulcus, Calc S: calcarine sulcus) is indicated. Source data are provided as a Source Data file.

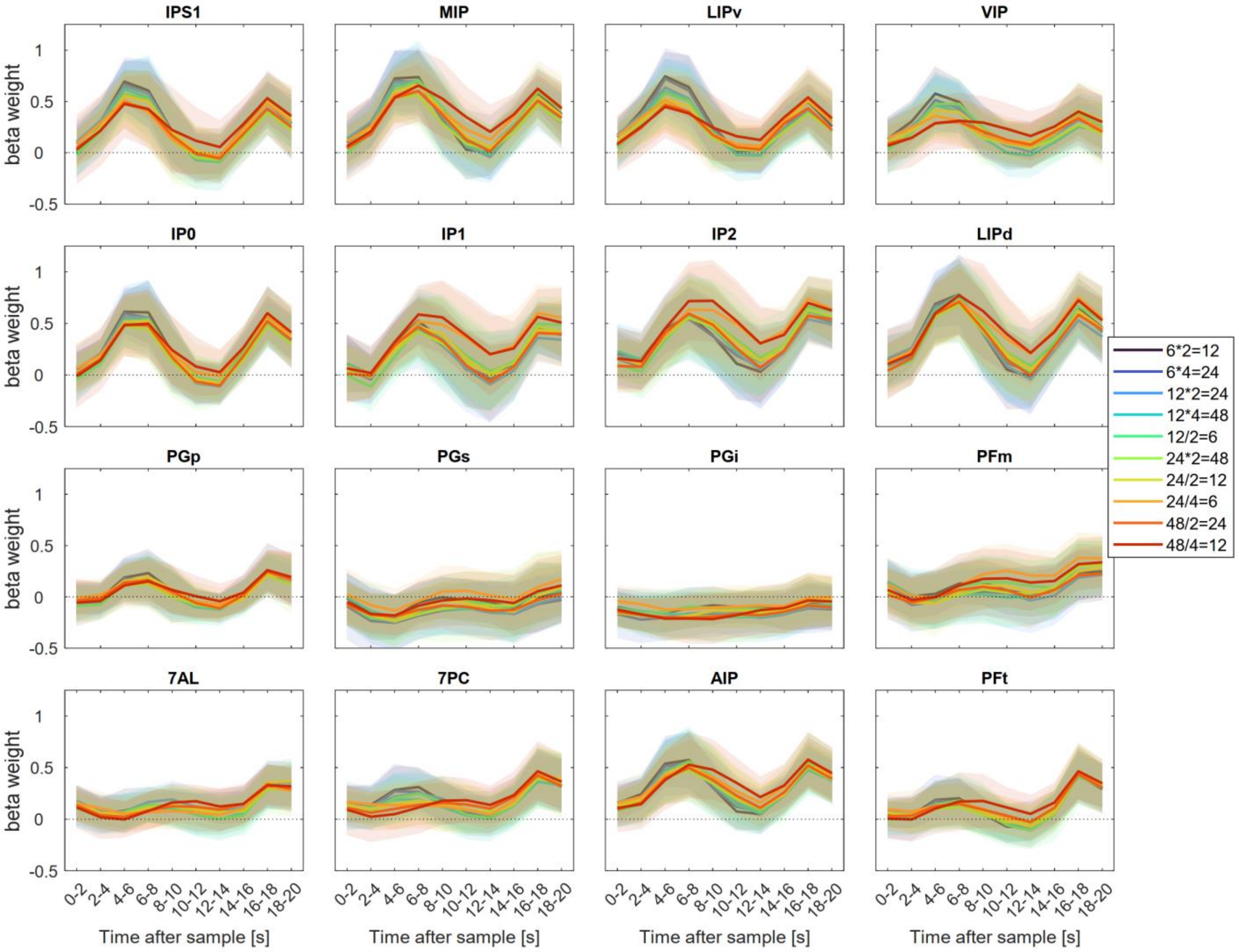


**Supplementary Figure 3: Evoked activity time courses as computed by FIR estimates for all parietal regions of interest.** Means and standard deviations across subjects (N=17) are shown for the 10 experimental conditions. Source data are provided as a Source Data file.

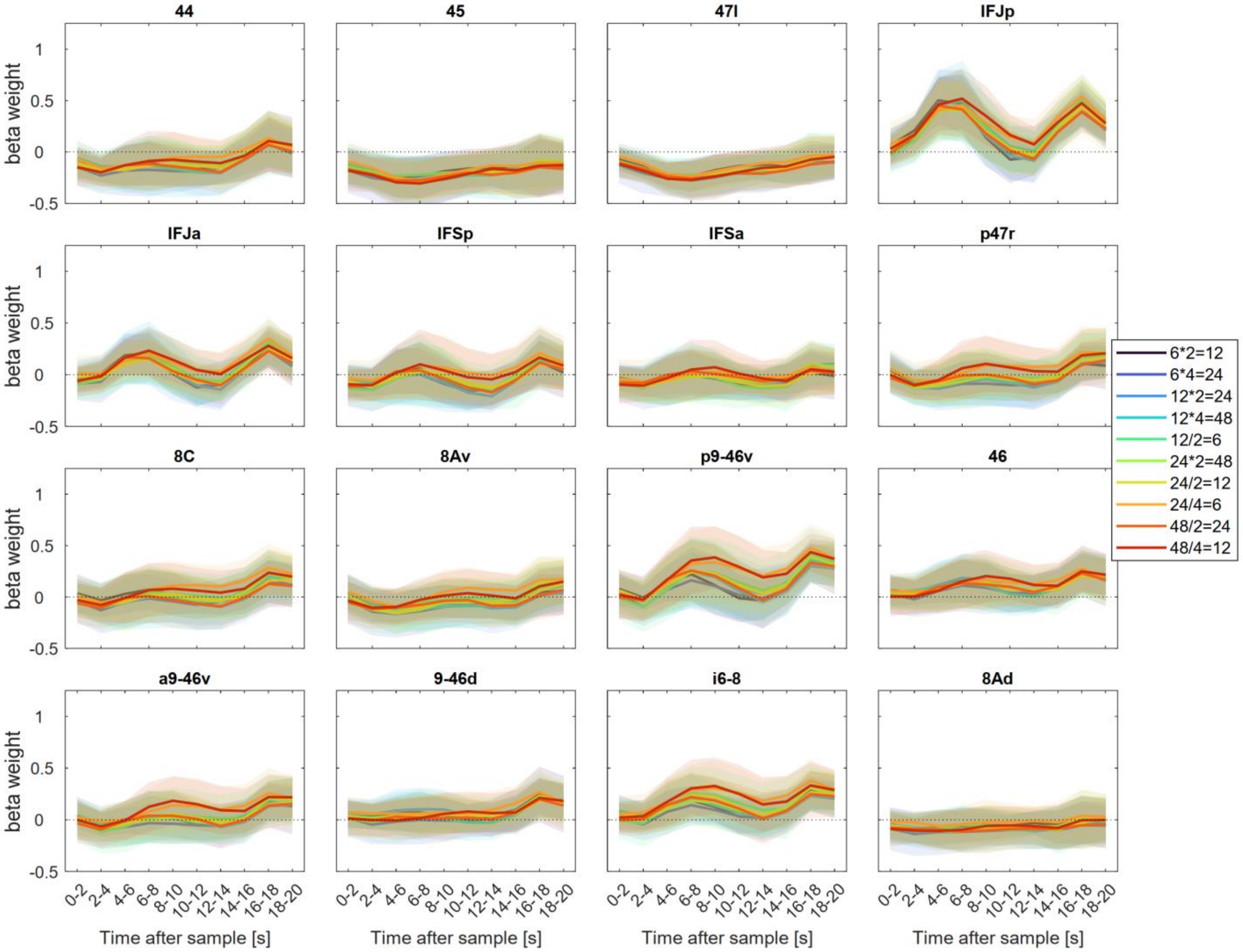


**Supplementary Figure 4: Evoked activity time courses as computed by FIR estimates for all frontal regions of interest.** Means and standard deviations across subjects (N=17) are shown for the 10 experimental conditions. Source data are provided as a Source Data file.

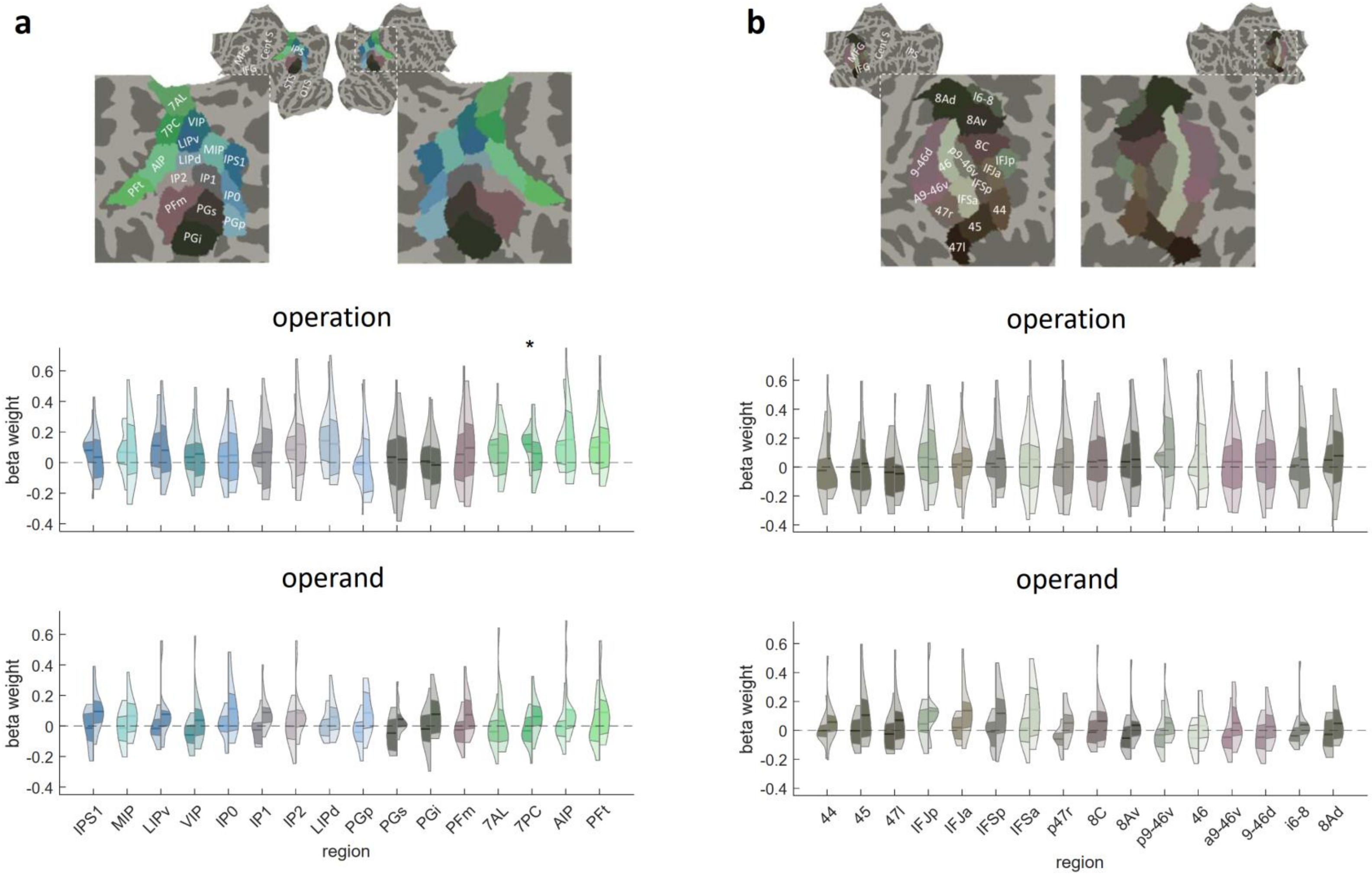


**Supplementary Figure 5: Effects of operation and operand in representational similarity analysis on regions of interest (ROIs).** Results for operation and operand predictors in multiple regression analyses on brain activity pattern dissimilarity are shown for subregions of parietal (A) and lateral prefrontal (B) cortex. ROI delineation, colors and labels are based on the Human Connectome Project (HCP-MMP1) parcellation shown here on the flattened cortical average surface. For each ROI, violin plots show the distribution of beta estimates for operation and operand predictors (n = 17 subjects). The central horizontal line indicates the mean, and the adjacent colored area the limits of the first and third quartile, with the left part of the violin for each ROI corresponding to averages of the middle (4-8 s), and the right part to averages of the late (8-12 s) delay period. Stars indicate significantly larger responses than 0 in two-tailed t-tests, after FDR

correction for multiple comparisons (* corresponds to pFDR<.05, across 32 subregions and two time periods). The only effect significant was in 7PC for the operation for middle time bins (t(16) = 5.11, p = .0001, pFDR = .007). Source data are provided as a Source Data file.

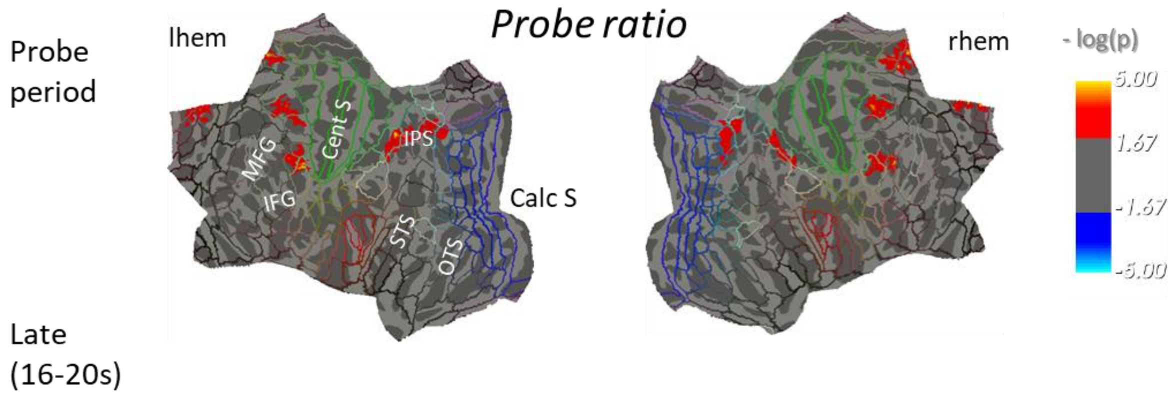


**Supplementary Figure 6: Differential effects of close > far probe ratio.** Results of surface based group analysis (n=17 subjects) are shown for the late probe interval (two-tailed one-sample t-test, corrected for multiple comparisons by permutation at cluster level, pFWE <.05, cluster forming threshold $p < .01$). The color scale represents uncorrected -log(p) values within the clusters surviving correction. Results are projected onto a flattened cortical average surface (https://mri.sdsu.edu/sereno/csurf/), with superimposed lines representing the regional borders of the Human Connectome Project (HCP-MMP1) parcellation. For better orientation, the location of major anatomical landmarks (MFG: middle frontal gyrus, IFG: inferior frontal gyrus, Cent S: central sulcus, IPS: intraparietal sulcus, STS: superior temporal sulcus, OTS: occipitotemporal sulcus, Calc S: calcarine sulcus) is indicated. Source data are provided as a Source Data file.

## Supplementary Tables

Supplementary Table 1: Cluster summary table for a surface-based group analysis (N=17) conducted on univariate effects of sample and result numerosity when modelling responses by a canonical HRF.

For each contrast displayed in Supplementary Figure 1, and each cluster surviving $p_{FWE} < .05$ (corrected at cluster level by permutation methods with cluster forming threshold $p < .01$) the table reports: the cluster label for the activation maximum (as defined by the anatomical labels from the Destrieux Atlas), the maximum -log10(p) value in the cluster (Max), the cluster surface area in $mm^2$ (Size), the MNI coordinates of the maximally activated vertex within each cluster (MNI X, Y, Z), the cluster-wise p-value of each cluster (CWP), the number of vertices included in each cluster (NVtxs).

| Hemisphere | Cluster Label | Max | Size ($mm^2$) | MNI X | MNI Y | MNI Z | CWP | NVtxs |
|---|---|---|---|---|---|---|---|---|
| | | | Sample parametric increase | | | | | |
| Left | G_oc-temp_med-Lingual | 7.08 | 2416.5 | -8.1 | -93.3 | -10.2 | 0.0004 | 2883 |
| | G_parietal_sup | -6.86 | 1395.1 | -29.4 | -55.7 | 56 | 0.0060 | 3253 |
| Right | S_intrapariet_and_P_trans | -6.21 | 2804.6 | 34.5 | -46.4 | 49.1 | 0.0008 | 6100 |
| | Pole_occipital | 7.62 | 1936.9 | 14.4 | -97.4 | 4.3 | 0.0024 | 2341 |
| | G_and_S_occipital_inf | -3.59 | 637.4 | 45.4 | -64.3 | -11.3 | 0.0290 | 874 |
| | | | Sample 6 > other | | | | | |
| Left | G_oc-temp_med-Lingual | -6.36 | 2078.2 | -8.1 | -92.8 | -10.4 | 0.0024 | 2461 |
| | G_parietal_sup | 5.71 | 696.6 | -28.9 | -55.7 | 55.4 | 0.0183 | 1598 |
| Right | Pole_occipital | -5.97 | 1599.8 | 22.2 | -99.5 | 0.3 | 0.0028 | 1952 |
| | S_intrapariet_and_P_trans | 6.21 | 1511.9 | 25.4 | -55.5 | 56.7 | 0.0036 | 3568 |
| | | | Sample 48 > other | | | | | |
| Left | G_oc-temp_med-Lingual | 6.29 | 2135.2 | -17.9 | -86.1 | -9.5 | 0.0016 | 2516 |
| | G_parietal_sup | -6.68 | 1481.1 | -30.1 | -55.6 | 56.3 | 0.0024 | 3458 |
| | S_precentral-sup-part | -5.97 | 420.5 | -22.5 | -8.4 | 48.2 | 0.0400 | 893 |
| | S_intrapariet_and_P_trans | -5.34 | 407.9 | -25.4 | -70.3 | 23 | 0.0423 | 747 |
| Right | S_intrapariet_and_P_trans | -6.17 | 2666.2 | 25.3 | -58.8 | 47.7 | 0.0004 | 5834 |
| | Pole_occipital | 5.59 | 1404.7 | 14.8 | -94.8 | 4.7 | 0.0044 | 1713 |
| | S_precentral-sup-part | -5.17 | 458.3 | 31.8 | -9.7 | 48.9 | 0.0373 | 1056 |
| | | | Result 6 > other | | | | | |
| Right | G_pariet_inf-Angular | 5.15 | 766.5 | 53.2 | -52.1 | 30.8 | 0.01196 | 1568 |
| | | | Result 24 > other | | | | | |
| Left | S_front_inf | -4.60 | 543.3 | -40.2 | 21.5 | 30.2 | 0.0262 | 1006 |
| Right | G_pariet_inf-Angular | -5.08 | 541.8 | 38.7 | -73.6 | 33.3 | 0.0317 | 902 |

Supplementary Table 2: Cluster summary table for differential contrasts between different parts of the delay period for the surface-based group analysis (n=17 subjects) conducted on the searchlight pattern analysis (multiple regression with sample, operation, operand and result predictors on the fMRI distance matrices).

For each contrast displayed in Supplementary Figure 2, and each cluster surviving $p_{FWE} < .05$ in a two-tailed one-sample t-test (corrected at cluster level by permutation methods with cluster forming threshold $p < .01$) the table reports: the cluster label for the activation maximum (as defined by the anatomical labels from the Destrieux Atlas), the maximum -log10(p) value in the cluster (Max), the cluster surface area in $mm^2$ (Size), the MNI coordinates of the maximally activated vertex within each cluster (MNI X, Y, Z), the cluster-wise p-value of each cluster (CWP), the number of vertices included in each cluster (NVtxs). No above threshold clusters were detected for the operation and the operand at any time window.

| Hemisphere | Cluster Label | Max | Size ($mm^2$) | MNI X | MNI Y | MNI Z | CWP | NVtxs |
|---|---|---|---|---|---|---|---|---|
| | | | Sample – middle > early delay | | | | | |
| Left | S_oc_sup_and_transversal | 5.55 | 2700.1 | -34.3 | -79.8 | 18.4 | 0.0020 | 4573 |
| Right | G_occipital_middle | 5.96 | 5932.8 | 36.5 | -82.5 | 20.9 | 0.0004 | 11367 |
| | S_front_sup | 4.57 | 910.7 | 27.4 | 1.7 | 48.9 | 0.0243 | 1745 |
| | S_precentral-inf-part | 4.02 | 878.1 | 49.0 | 9.9 | 12.2 | 0.0270 | 1674 |
| | | | Sample – late > early delay | | | | | |
| Right | G_temporal_middle | 4.78 | 669.8 | 61.1 | -46.9 | 0.5 | 0.0459 | 1157 |
| | | | Sample – late > middle delay | | | | | |
| Right | G_parietal_sup | - 4.24 | 503.9 | 26.6 | -54.8 | 57.7 | 0.0490 | 1109 |
| | | | Sample – late and middle > early delay | | | | | |
| Left | G_occipital_sup | 4.00 | 1249.6 | -21.5 | -80.5 | 33.1 | 0.0183 | 2194 |
| Right | S_intrapariet_and_P_trans | 4.15 | 2082.8 | 20.2 | -59.9 | 52.0 | 0.0060 | 3956 |
| | G_pariet_inf-Angular | 4.49 | 852.7 | 46.7 | -53.7 | 44.0 | 0.0317 | 2123 |
| | G_front_sup | 5.02 | 846.7 | 9.1 | 11.7 | 56.4 | 0.0321 | 1689 |
| | | | Result – middle > early delay | | | | | |
| Left | S_temporal_sup | 5.14 | 459.2 | -40.1 | -63.9 | 33.0 | 0.0388 | 919 |
| | | | Result – late > early delay | | | | | |
| Left | G_pariet_inf-Angular | 5.11 | 1543.5 | -37.9 | -72.5 | 36.4 | 0.0120 | 3423 |
| | S_subparietal | 5.02 | 872.9 | -7.4 | -48.8 | 29.6 | 0.0373 | 1812 |
| | G_front_middle | 4.56 | 794.5 | -40.1 | 10.9 | 50.7 | 0.0463 | 1398 |
| | | | Result – middle and late > early delay | | | | | |
| Left | S_front_sup | 4.20 | 1020.0 | -27.1 | 31.9 | 28.1 | 0.0207 | 1760 |
| | S_subparietal | 5.04 | 881.7 | -10.5 | -57.2 | 29.9 | 0.0258 | 1828 |
| | S_temporal_sup | 5.40 | 822.3 | -40.3 | -63.6 | 33.4 | 0.0298 | 1729 |

Supplementary Table 3: Results (n=17 subjects) of cross-decoding performance between sample and result numerosity representations for the 16 parietal and 16 frontal ROIs derived from the HCP-MMP1 parcellation. For each ROI, the mean of the decoding performance corresponding to a correlation score (mean) is reported together with effect size (Cohen's d), t-value of two-tailed t-tests against 0, degrees of freedom, uncorrected p values, lower and upper bound of the 95% confidence interval around the mean, and corrected p values. Significance was corrected for multiple comparisons across all ROIs by false discovery rate (FDR).

| ROI | mean | Cohen's d | t | df | $p_{uncorr}$ | $CI_l$ | $CI_u$ | $p_{FDR\ corr}$ |
|---|---|---|---|---|---|---|---|---|
| IPS1 | 0.043 | 0.433 | 1.88 | 16 | 0.0789 | 0.003 | 0.084 | 0.6315 |
| MIP | 0.055 | 0.593 | 2.57 | 16 | 0.0206 | 0.018 | 0.092 | 0.5580 |
| LIPv | 0.030 | 0.296 | 1.28 | 16 | 0.2188 | -0.011 | 0.071 | 0.7862 |
| VIP | 0.017 | 0.214 | 0.93 | 16 | 0.3685 | -0.015 | 0.050 | 0.7862 |
| IP0 | 0.038 | 0.484 | 2.10 | 16 | 0.0523 | 0.006 | 0.069 | 0.5580 |
| IP1 | 0.017 | 0.228 | 0.99 | 16 | 0.3389 | -0.013 | 0.047 | 0.7862 |
| IP2 | 0.004 | 0.046 | 0.20 | 16 | 0.8454 | -0.033 | 0.042 | 0.9360 |
| LIPd | 0.006 | 0.069 | 0.30 | 16 | 0.7674 | -0.028 | 0.039 | 0.9360 |
| PGp | 0.019 | 0.335 | 1.45 | 16 | 0.1664 | -0.004 | 0.043 | 0.7605 |
| PGs | 0.012 | 0.117 | 0.51 | 16 | 0.6195 | -0.030 | 0.055 | 0.9360 |
| PGi | 0.024 | 0.218 | 0.94 | 16 | 0.3596 | -0.020 | 0.068 | 0.7862 |
| PFm | -0.006 | -0.063 | 0.27 | 16 | 0.7875 | -0.041 | 0.030 | 0.9360 |
| 7AL | 0.015 | 0.166 | 0.72 | 16 | 0.4837 | -0.022 | 0.052 | 0.9104 |
| 7PC | 0.005 | 0.061 | 0.27 | 16 | 0.7940 | -0.028 | 0.038 | 0.9360 |
| AIP | 0.021 | 0.235 | 1.02 | 16 | 0.3243 | -0.015 | 0.057 | 0.7862 |
| PFt | 0.020 | 0.231 | 1.00 | 16 | 0.3315 | -0.015 | 0.056 | 0.7862 |
| 44 | 0.018 | 0.231 | 1.00 | 16 | 0.3323 | -0.014 | 0.050 | 0.7862 |
| 45 | 0.003 | 0.035 | 0.15 | 16 | 0.8817 | -0.027 | 0.032 | 0.9360 |
| 47l | 0.012 | 0.154 | 0.67 | 16 | 0.5145 | -0.019 | 0.042 | 0.9146 |
| IFJp | 0.022 | 0.377 | 1.63 | 16 | 0.1219 | -0.002 | 0.046 | 0.6500 |
| IFJa | -0.005 | -0.095 | 0.41 | 16 | 0.6867 | -0.027 | 0.017 | 0.9360 |
| IFSp | 0.026 | 0.381 | 1.65 | 16 | 0.1189 | -0.002 | 0.053 | 0.6500 |
| IFSa | 0.008 | 0.141 | 0.61 | 16 | 0.5512 | -0.015 | 0.031 | 0.9283 |
| p47r | 0.018 | 0.266 | 1.15 | 16 | 0.2655 | -0.009 | 0.046 | 0.7862 |
| 8C | 0.001 | 0.007 | 0.03 | 16 | 0.9772 | -0.036 | 0.037 | 0.9772 |
| 8Av | 0.002 | 0.028 | 0.12 | 16 | 0.9038 | -0.027 | 0.031 | 0.9360 |
| p9-46v | 0.004 | 0.050 | 0.22 | 16 | 0.8303 | -0.027 | 0.035 | 0.9360 |
| 46 | 0.010 | 0.126 | 0.55 | 16 | 0.5923 | -0.023 | 0.044 | 0.9360 |
| a9-46v | -0.002 | -0.027 | 0.12 | 16 | 0.9068 | -0.037 | 0.032 | 0.9360 |
| 9-46d | -0.012 | -0.198 | 0.86 | 16 | 0.4042 | -0.035 | 0.012 | 0.8084 |
| i6-8 | 0.036 | 0.499 | 2.16 | 16 | 0.0463 | 0.007 | 0.065 | 0.5580 |
| 8Ad | -0.004 | -0.039 | 0.17 | 16 | 0.8682 | -0.041 | 0.034 | 0.9360 |

**Supplementary Table 4:** Results (n=17 subjects) of the correlation analysis between cross-decoding performance between sample and result numerosity representations and behavioral Weber fractions, for the 16 parietal and 16 frontal ROIs derived from the HCP-MMP1 parcellation. For each ROI, the Pearson correlation coefficient (r) is reported together with uncorrected as well as corrected p values, and the lower and upper bound of the 95% confidence interval of the correlation coefficient. Significance was corrected for multiple comparisons across all ROIs by false discovery rate (FDR).

| ROI | r | $p_{uncorr}$ | $p_{FDR\ corr}$ | $CI_l$ | $CI_u$ | ROI | rho | $p_{uncorr}$ | $CI_l$ | $CI_u$ | $p_{FDR\ corr}$ |
|---|---|---|---|---|---|---|---|---|---|---|---|
| IPS1 | -0.23 | 0.3852 | 0.8632 | -0.64 | 0.29 | 44 | 0.29 | 0.2585 | -0.22 | 0.68 | 0.8632 |
| MIP | -0.80 | 0.0001 | 0.0035 | -0.93 | -0.52 | 45 | -0.16 | 0.5398 | -0.59 | 0.35 | 0.8632 |
| LIPv | -0.37 | 0.1442 | 0.8632 | -0.72 | 0.13 | 47l | -0.22 | 0.3875 | -0.64 | 0.29 | 0.8632 |
| VIP | -0.06 | 0.8190 | 0.9719 | -0.53 | 0.43 | IFJp | -0.16 | 0.5326 | -0.60 | 0.34 | 0.8632 |
| IP0 | -0.18 | 0.5006 | 0.8632 | -0.61 | 0.33 | IFJa | -0.17 | 0.5045 | -0.60 | 0.33 | 0.8632 |
| IP1 | -0.12 | 0.6474 | 0.8632 | -0.57 | 0.38 | IFSp | -0.23 | 0.3649 | -0.64 | 0.28 | 0.8632 |
| IP2 | -0.06 | 0.8272 | 0.9719 | -0.52 | 0.44 | IFSa | -0.12 | 0.6415 | -0.57 | 0.38 | 0.8632 |
| LIPd | -0.13 | 0.6063 | 0.8632 | -0.58 | 0.37 | p47r | -0.46 | 0.0647 | -0.77 | 0.03 | 0.8632 |
| PGp | -0.05 | 0.8576 | 0.9719 | -0.52 | 0.44 | 8C | 0.01 | 0.9583 | -0.47 | 0.49 | 0.9875 |
| PGs | -0.36 | 0.1515 | 0.8632 | -0.72 | 0.14 | 8Av | -0.22 | 0.3910 | -0.64 | 0.29 | 0.8632 |
| PGi | -0.23 | 0.3746 | 0.8632 | -0.64 | 0.28 | p9-46v | 0.25 | 0.3390 | -0.27 | 0.65 | 0.8632 |
| PFm | -0.25 | 0.3253 | 0.8632 | -0.65 | 0.26 | 46 | -0.08 | 0.7625 | -0.54 | 0.42 | 0.9719 |
| 7AL | -0.17 | 0.5140 | 0.8632 | -0.60 | 0.34 | a9-46v | -0.25 | 0.3362 | -0.65 | 0.26 | 0.8632 |
| 7PC | -0.19 | 0.4637 | 0.8632 | -0.61 | 0.32 | 9-46d | -0.12 | 0.6432 | -0.57 | 0.38 | 0.8632 |
| AIP | 0.04 | 0.8808 | 0.9719 | -0.45 | 0.51 | i6-8 | 0.00 | 0.9875 | -0.48 | 0.48 | 0.9875 |
| PFt | -0.19 | 0.4671 | 0.8632 | -0.61 | 0.32 | 8Ad | -0.02 | 0.9429 | -0.49 | 0.47 | 0.9875 |

Supplementary Table 5: Cluster summary table for the surface-based group analysis (n=17 subjects) conducted on the univariate contrast of probe ratio (close > far).

For the contrast displayed in Supplementary Figure 6, and each cluster surviving $p_{FWE} < .05$ in a two-tailed one-sample t-test (corrected at cluster level by permutation methods with cluster forming threshold $p < .01$) the table reports: the cluster label for the activation maximum (as defined by the anatomical labels from the Destrieux Atlas), the maximum -log10(p) value in the cluster (Max), the cluster surface area in $mm^2$ (Size), the MNI coordinates of the maximally activated vertex within each cluster (MNI X, Y, Z), the cluster-wise p-value of each cluster (CWP), and the number of vertices included in each cluster (NVtxs). No above threshold clusters were detected for the early, middle and late delay period, nor for the early probe period.

| Hemisphere | Cluster Label | Max | Size ($mm^2$) | MNI X | MNI Y | MNI Z | CWP | NVtxs |
|---|---|---|---|---|---|---|---|---|
| | | | Ratio (close > far) – late probe period | | | | | |
| Left | S_intrapariet_and_P_trans | 5.46 | 513.0 | -33 | -46.6 | 44 | 0.0223 | 1408 |
| | G_and_S_cingul-Mid-Ant | 4.89 | 465.4 | -10.8 | 15.7 | 43 | 0.0302 | 900 |
| | S_intrapariet_and_P_trans | 3.76 | 416.6 | -21.5 | -72.3 | 34.8 | 0.0357 | 857 |
| | G_precentral | 5.95 | 406.5 | -53.6 | 4.7 | 25.9 | 0.0376 | 869 |
| | S_front_sup | 4.18 | 404.3 | -21.1 | -4.6 | 48.3 | 0.0384 | 890 |
| Right | G_and_S_cingul-Mid-Ant | 5.2302 | 756.59 | 13.9 | 25.4 | 27.8 | 0.0148 | 1588 |
| | S_front_inf | 5.2304 | 510.1 | 37.5 | 21 | 22.7 | 0.0282 | 1149 |
| | S_intrapariet_and_P_trans | 3.8723 | 493.39 | 18.4 | -66.2 | 47.6 | 0.0306 | 858 |
| | S_precentral-sup-part | 4.5081 | 448.14 | 24.4 | -6.1 | 45.5 | 0.0357 | 1032 |
| | S_intrapariet_and_P_trans | 3.7602 | 417.24 | 31.9 | -41.2 | 38.5 | 0.0400 | 1106 |
