# Supplementary material for: Human brain representations of internally generated outcomes of approximate calculation revealed by ultra-high-field brain imaging
Source: Nat Commun. 2024 Jan 17;15:572. doi: 10.1038/s41467-024-44810-5 (PMC10794709; doi:10.1038/s41467-024-44810-5)
Supplement: Supplementary file 3 — Reporting Summary [file 41467_2024_44810_MOESM3_ESM.pdf]

nature portfolio

Corresponding author(s): Evelyn Eger

Last updated by author(s): Dec 5, 2023

Reporting Summary

Nature Portfolio wishes to improve the reproducibility of the work that we publish. This form provides structure for consistency and transparency in reporting. For further information on Nature Portfolio policies, see our Editorial Policies and the Editorial Policy Checklist.

Statistics

For all statistical analyses, confirm that the following items are present in the figure legend, table legend, main text, or Methods section.

n/a	Confirmed	
☐	☒	The exact sample size (*n*) for each experimental group/condition, given as a discrete number and unit of measurement
☐	☒	A statement on whether measurements were taken from distinct samples or whether the same sample was measured repeatedly
☐	☒	The statistical test(s) used AND whether they are one- or two-sided<br>*Only common tests should be described solely by name; describe more complex techniques in the Methods section.*
☐	☒	A description of all covariates tested
☐	☒	A description of any assumptions or corrections, such as tests of normality and adjustment for multiple comparisons
☐	☒	A full description of the statistical parameters including central tendency (e.g. means) or other basic estimates (e.g. regression coefficient) AND variation (e.g. standard deviation) or associated estimates of uncertainty (e.g. confidence intervals)
☐	☒	For null hypothesis testing, the test statistic (e.g. *F*, *t*, *r*) with confidence intervals, effect sizes, degrees of freedom and *P* value noted<br>*Give P values as exact values whenever suitable.*
☒	☐	For Bayesian analysis, information on the choice of priors and Markov chain Monte Carlo settings
☒	☐	For hierarchical and complex designs, identification of the appropriate level for tests and full reporting of outcomes
☐	☒	Estimates of effect sizes (e.g. Cohen's *d*, Pearson's *r*), indicating how they were calculated

Our web collection on statistics for biologists contains articles on many of the points above.

Software and code

Policy information about availability of computer code

Data collection	Siemens Magnetom 7T scanner, Psychophysics Toolbox 3
Data analysis	Freesurfer 6.0 (https://surfer.nmr.mgh.harvard.edu/), FSL 5.0 (https://fsl.fmrib.ox.ac.uk/fsl/fslwiki/FSL), SPM12 (https://www.fil.ion.ucl.ac.uk/spm/software/spm12/), CoSMoMVPA (https://www.cosmomvpa.org/), Matlab version 2019b (The Mathworks), Python 3, Nilearn (https://nilearn.github.io/stable/index.html), scikit-learn (https://scikit-learn.org/stable/)

For manuscripts utilizing custom algorithms or software that are central to the research but not yet described in published literature, software must be made available to editors and reviewers. We strongly encourage code deposition in a community repository (e.g. GitHub). See the Nature Portfolio guidelines for submitting code & software for further information.

Data

Policy information about availability of data

All manuscripts must include a data availability statement. This statement should provide the following information, where applicable:

- Accession codes, unique identifiers, or web links for publicly available datasets
- A description of any restrictions on data availability
- For clinical datasets or third party data, please ensure that the statement adheres to our policy

The functional imaging data (individual subjects' first-level fMRI models, anatomical images, and ROI images) generated as part of this study have been deposited in an Open Science Framework database (https://osf.io/2tnrz/). Source data plotted in the figures are provided with this paper.

Research involving human participants, their data, or biological material

Policy information about studies with human participants or human data. See also policy information about sex, gender (identity/presentation), and sexual orientation and race, ethnicity and racism.

Reporting on sex and gender	Participants of both female and male sex (according to self report) were included in the study. No additional information on gender was collected. Sex and/or gender was not considered as a factor of interest in the design of the study, as this study was interested in brain correlates of numerical processing in as far as they apply to normal adult human subjects in general. As a consequence, the analyses reported in the paper concern the combined group of all subjects (which was roughly balanced for males and females). Separate analyses as a function of sex were not performed, since the resulting sample sizes of the individual groups are judged to be not large enough.
Reporting on race, ethnicity, or other socially relevant groupings	The constructs of race/ethnicity or other socially relevant grouping were not considered in our study design, nor was any information related to them collected. These factors are not considered relevant for the questions related to cognition and brain function which are in the focus of this work.
Population characteristics	The collected sample included healthy adults aged 25.9 +/- 6.9 years old.
Recruitment	Subjects were recruited by para-medical staff of the host institution. More specifically, the neuroimaging research done at Neurospin is regularly advertised in surrounding universities and other public institutions. Persons interested in participating can then contact the para-medical staff who perform a first check to exclude contra-indications for participation in MRI research, after which they are included in a local volunteer database, and can inscribe themselves for proposed slots of ongoing experiments. Since the decision to participate is left up to the individual, self selection bias is likely present (yielding participants with a higher degree of interest in the topic of the study or brain function more generally). These motivational factors are, however, unlikely to affect brain activity differences in response to the stimuli presented, and thus the pattern of results obtained.
Ethics oversight	Comité de protection des personnes [CPP] Ile de France VII, Hôpital de Bicêtre

Note that full information on the approval of the study protocol must also be provided in the manuscript.

Field-specific reporting

Please select the one below that is the best fit for your research. If you are not sure, read the appropriate sections before making your selection.

☐ Life sciences ☒ Behavioural & social sciences ☐ Ecological, evolutionary & environmental sciences

For a reference copy of the document with all sections, see nature.com/documents/nr-reporting-summary-flat.pdf

Behavioural & social sciences study design

All studies must disclose on these points even when the disclosure is negative.

Study description	quantitative cross-sectional (fMRI group study)
Research sample	We acquired fMRI data in 18 subjects (9 male and 9 female, 25.9 +/- 6.9 years old), with normal visual acuity, free of neurological or psychiatric disease, and without a history of learning disability, representative of well-educated young adults from the greater Paris area. The rationale for selecting a sample of healthy, neurotypical participants is that we are interested in the study or normal brain function.
Sampling strategy	The group represents a convenience sample of healthy young adults inscribed in the volunteer database of the host institution, who could sign up for the proposed experimental slots on the internet. No a priori sample size calculation was performed, but the sample size was chosen to be comparable to previous studies of our group in the domain of interest at the same MRI field strength (e.g., Castaldi et al., 2019, Elife, Castaldi et al., 2020).
Data collection	Data were collected on a SIEMENS Magnetom 7 T scanner. A radiographer was present in addition to the researcher in the console room while the participant was in the scanner. Researchers were not blinded (which is irrelevant since experimental conditions were manipulated within subject and their presentation controlled by stimulus delivery software in an automatized fashion).
Timing	Data were collected between 01/04/2019 and 01/08/2019.
Data exclusions	Data of one participant were excluded from analysis after failing to properly understand task instructions and pressing response buttons erratically while in the scanner.
Non-participation	No participant dropped out / declined participation.
Randomization	Participants were allocated to a single group. Experimental conditions were presented in randomized order within each scanning session (randomized separately for each participant).

Reporting for specific materials, systems and methods

We require information from authors about some types of materials, experimental systems and methods used in many studies. Here, indicate whether each material, system or method listed is relevant to your study. If you are not sure if a list item applies to your research, read the appropriate section before selecting a response.

Materials & experimental systems

n/a	Involved in the study
☒	☐ Antibodies
☒	☐ Eukaryotic cell lines
☒	☐ Palaeontology and archaeology
☒	☐ Animals and other organisms
☒	☐ Clinical data
☒	☐ Dual use research of concern
☒	☐ Plants

Methods

n/a	Involved in the study
☒	☐ ChIP-seq
☒	☐ Flow cytometry
☐	☒ MRI-based neuroimaging

Plants

Seed stocks: n.a.

Novel plant genotypes: n.a.

Authentication: n.a.

Magnetic resonance imaging

Experimental design

Design type: event-related design

Design specifications: There were 10 main trial types consisting of different combinations of sample number presentation, operation cue, delay period and probe number presentation. Each experimental run included two trials of each of the 10 main trial types, and subjects completed between 6 and 8 runs. The SOA between sample and probe number was 12 s. It was unpredictably shortened on an additional 20 % of trials (catch trials, not considered in the analysis). The SOA between the start of one trial and the next trial was 20 s.

Behavioral performance measures: correct button presses, reported as means and standard deviation across subjects

Acquisition

Imaging type(s): functional, structural

Field strength: 7 Tesla

Sequence & imaging parameters: multiband gradient echo EPI (multi-band [MB] = 2, GRAPPA acceleration with [IPAT] = 2, partial Fourier [PF] = 7/8, matrix = 130x130, repetition time [TR] = 2 s, echo time [TE] = 22 ms, echo spacing [ES] = 0.64 ms, flip angle [FA] = 68°, bandwidth [BW] = 1832Hz/Px, phase-encode direction anterior >> posterior), MP2Rage (GRAPPA acceleration with [IPAT] = 3, partial Fourier [PF] = 6/8, matrix = 256 x 256, repetition time [TR] = 5 s, echo time [TE] = 2.84 ms, time of inversion [TI] 1/2 = 800/2700 ms, flip angle [FA] 1/2 = 4°/5°, bandwidth [BW] = 240 Hz/px)

Area of acquisition: 68 oblique slices covering the occipito-temporal, parietal and frontal cortex, excluding cerebellum

Diffusion MRI ☐ Used ☒ Not used

Preprocessing

Preprocessing software: motion correction (SPM12), Topup distortion correction (FSL), cortical surface reconstruction (Freesurfer 6.0), boundary based registration (FreeSurfer 6.0), surface-constrained smoothing FWHM 2 mm (Freesurfer 6.0)

Normalization	Non-linear normalization in surface space (FreeSurfer)
Normalization template	Fsaverage
Noise and artifact removal	motion parameters as covariates of no interest in GLM
Volume censoring	None

Statistical modeling & inference

Model type and settings	First-level fixed-effects model within subjects, RSA analysis within subject, second-level random-effects group statistics
Effect(s) tested	RSA analysis: effects of predictors corresponding to pairwise differences in sample number, operation, operand, and result number across the 10 experimental conditions, regressed onto fMRI pattern distance matrices in multiple regression

Specify type of analysis: ☐ Whole brain ☐ ROI-based ☒ Both

Anatomical location(s): HCP-MMP1 atlas

Statistic type for inference (See Eklund et al. 2016)	cluster-wise inference (pFWE < .05, with cluster forming threshold p < .01)
Correction	FWE correction by permutation in whole brain analyses, FDR correction in ROI analysis

Models & analysis

n/a	Involved in the study
☒	☐ Functional and/or effective connectivity
☒	☐ Graph analysis
☐	☒ Multivariate modeling or predictive analysis

Multivariate modeling and predictive analysis	Multivariate decoding by linear support vector regression (C=1), applied to patterns extracted from ROIs without further feature selection, cross-validation with leave-one-session-out, evaluation of performance by Pearson correlation score between real and predicted labels.
